# Mutation characteristics of cancer susceptibility genes in Chinese ovarian cancer patients

**DOI:** 10.3389/fonc.2024.1395818

**Published:** 2024-05-16

**Authors:** Jie Wang, Kaiyu Fu, Mengpei Zhang, Lunggang Liang, Meng Ni, Hai-Xi Sun, Rutie Yin, Meifang Tang

**Affiliations:** ^1^ College of Life Sciences, University of Chinese Academy of Sciences, Beijing, China; ^2^ BGI Genomics, Shenzhen, China; ^3^ Department of Obstetrics and Gynecology, West China Second University Hospital, Sichuan University, Chengdu, Sichuan, China; ^4^ Laboratory of Molecular Epidemiology of Birth Defects, West China Second University Hospital, Sichuan University, Chengdu, Sichuan, China; ^5^ BGI Research, Beijing, China

**Keywords:** cancer genomics, cancer susceptibility genes, target capture sequencing, germline mutations, ovarian cancer

## Abstract

**Introduction:**

The association between mutations in susceptibility genes and the occurrence of ovarian cancer has been extensively studied. Previous research has primarily concentrated on genes involved in the homologous recombination repair pathway, particularly *BRCA1* and *BRCA2*. However, a wider range of genes related to the DNA damage response pathways has not been fully explored.

**Methods:**

To investigate the mutation characteristics of cancer susceptibility genes in the Chinese ovarian cancer population and the associations between gene mutations and clinical data, this study initially gathered a total of 1171 Chinese ovarian cancer samples and compiled a dataset of germline mutations in 171 genes.

**Results:**

In this study, it was determined that *MC1R* and *PRKDC* were high-frequency ovarian cancer susceptibility genes in the Chinese population, exhibiting notable distinctions from those in European and American populations; moreover high-frequency mutation genes, such as *MC1R*: c.359T>C and *PRKDC*: c.10681T>A, typically had high-frequency mutation sites. Furthermore, we identified c.8187G>T as a characteristic mutation of *BRCA2* in the Chinese population, and the *CHEK2* mutation was significantly associated with the early onset of ovarian cancer, while the *CDH1* and *FAM175A* mutations were more prevalent in Northeast China. Additionally, Fanconi anemia pathway-related genes were significantly associated with ovarian carcinogenesis.

**Conclusion:**

In summary, this research provided fundamental data support for the optimization of ovarian cancer gene screening policies and the determination of treatment, and contributed to the precise intervention and management of patients.

## Introduction

Ovarian cancer is a malignant gynecological tumor that seriously threatens the life and health of women. Among gynecological tumors worldwide, the incidence rate ranks third, and the mortality rate ranks first all year round. Global cancer statistics show that in 2020 (1), there were 313959 new cases of ovarian cancer and 207252 deaths worldwide. Among gynecological tumors in China (2), both incidence and mortality rank third, with more than 57090 new cases of ovarian cancer and 39306 deaths in 2020. By compiling cancer data in China in recent years (3), it can be found that the incidence rate of ovarian cancer is increasing annually.

Due to the special anatomical location of the ovary and the insidious onset of ovarian cancer, effective early screening methods are still lacking (4, 5). Therefore, 75% of patients are already in an advanced stage when diagnosed and have extensive intraperitoneal metastasis (6–8). Cancer susceptibility genes are significantly associated with ovarian carcinogenesis (9). In terms of family history, approximately 5-10% of patients with ovarian cancer have first-degree relatives with a history of ovarian cancer. Several studies have shown that, from the perspective of susceptibility genes, *BRCA1/2* is significantly associated with the occurrence of ovarian cancer (10). For example, women who have inherited *BRCA1* mutations have a lifetime risk of developing ovarian cancer ranging from 40% to 60%, while those with *BRCA2* mutations have a lifetime risk of 11% to 27% (11). A review of published patient data from the United States, the United Kingdom, and Australia found that in ovarian cancer (12), the frequency of *BRCA1* mutations in different countries ranged from 3.4% to 47%, and the frequency of *BRCA2* mutations ranged from 1% to 12%. A clinical study of ovarian cancer patients at Fujian Medical University Cancer Hospital revealed that 17.1% of patients carried *BRCA1* pathogenic mutations and 5.3% carried *BRCA2* pathogenic mutations (13); additionally, three Chinese-specific high-frequency *BRCA1* mutations, c.5470_5477delATTGGGCA, c.981_982delAT, c.3770 _3771delAG, were reported. Meanwhile, compared with those of individuals in the normal population, the risk ratio of individuals carrying *BRCA1* mutations was 34.6 for those aged younger than and 42.4 for those aged older than 50 years. A meta-analysis of published data from 1999 to 2017 (14), with technical platforms including PCR, Sanger sequencing, and high-throughput sequencing, included a total of 35178 cases of *BRCA1/2* testing in the Chinese population, of which the carrier rate of ovarian cancer was 21.8%. Owing to limitations in sample size, sampling regions, and differences in detection platforms, current studies can’t comprehensively and accurately profile the *BRCA1/2* mutations of ovarian cancer in the Chinese population.

DNA damage repair (DDR) refers to the cellular response in which damaged DNA molecules in cells maintain the relative stability of genetic information and restore the structure of normal DNA sequences through the cooperation of multiple proteins (15). The human cell has multiple mechanisms for DNA damage repair (16), such as homologous recombination repair (HRR), mismatch repair (MMR), Fanconi anemia, and base excision repair. Alterations in genes involved in DNA damage repair are closely associated with the occurrence, progression, and drug resistance of cancer (17). Poly (ADP-ribose) polymerase (PARP) inhibitors based on defects in the HRR pathway have been approved for marketing by the FDA. A study of 449 epithelial ovarian cancer gene mutations from Peking Union Medical College Hospital (18), including 28 HRR-related genes, 4 MMR genes, and 4 hereditary tumor-related genes, found that 107 patients carried *BRCA1/2* germline mutations, the other 31 patients were carriers of germline mutations in other DDR-related genes, and all *RAD51D* germline mutation carriers were patients younger than 40 years. A multigene germline mutation analysis of ovarian cancer patients at the University of Washington revealed that 18% of patients carried pathogenic mutations in susceptibility genes and that *PALB2* and *BARD1* were significantly associated with the occurrence of ovarian cancer (19). A survey initiated by Myriad Genetics, Inc., which enrolled patients undergoing genetic testing for hereditary tumor risk between 2013 and 2022, analyzed the relationship between pathogenic mutations and the occurrence of multiple cancers and revealed that *PTEN* pathogenic mutations resulted in a 3.77-fold increase in the risk of developing ovarian cancer (20). The impact of HRR-related genes on the occurrence of ovarian cancer has been widely researched. However, the mutations of other DNA damage repair genes in the Chinese ovarian cancer population and their relationship with patient characteristics have not been thoroughly investigated.

Clinical multigene testing, which can assess the risk of ovarian cancer, and provide data support for future cancer prevention, diagnosis, treatment, and optimal management, plays a crucial role in the treatment of ovarian cancer. In this study, we gathered data on multigene germline mutations from Chinese ovarian cancer patients and created a dataset of ovarian cancer germline mutations in the Chinese population. Secondly, based on the germline mutation characteristics of ovarian cancer in the Chinese population, the high-frequency mutations and genes in the Chinese population were analyzed. Finally, combined with family history, age of onset, and region, we analyzed and identified genes associated with family history, early onset of cancer, and geographical characteristics, and attempted to elucidate the mechanism between mutations and ovarian cancer occurrence.

## Materials and methods

### Sample selection and dataset construction

From the previously published literature, we searched for ovarian cancer studies that performed germline testing of 171 genes (**Supplementary Table S1**) at the BGI Shenzhen Clinical Diagnostic Laboratory. All samples were obtained with informed consent, and a total of 3 studies met the criteria (21–23). Mutation data was obtained from the authors based on a reasonable request. And mutation detection methods were described in detail in the **Supplementary Material 1**. The mutation results and clinical information from the three studies were pooled, and samples with missing age, family history of cancer, or regional information were excluded. The data of 1171 ovarian cancer samples that can represent ovarian cancer in the Chinese population were retained. The average depth was over 100X and the coverage at 30X exceeded 95% for each sample. The sequencing coverage and quality statistics for each sample were summarized in **Supplementary Table S2**.

### Mutation filtering and annotation

Single nucleotide variations (SNVs), and insertions and deletions (INDELs) were selected that were localized in all exons and intron-exon boundaries (± 20 bp) of the genes. Mutations with fewer than 5 supporting reads, a mutation frequency less than 20%, or a population frequency greater than 5% in the population polymorphism databases GnomAD, ExAC, and 1k Genomes were excluded (24–26). The final mutation results for each sample were obtained. According to the genetic variation classification standards and guidelines jointly developed by the American College of Medical Genetics and Genomics (ACMG) and the Association for Molecular Pathology (AMP) (27, 28), mutations were classified into five categories, pathogenic, likely pathogenic, variant of unknown significance (VUS), likely benign and benign. Patients with likely pathogenic or pathogenic mutations were defined as those carrying deleterious mutations.

### Statistical analysis

All the statistical analyses and plots were performed using R (version 3.6.3). Pearson’s χ2-test and Fisher’s exact test were used to determine the statistical significance of categorical variables. Student’s t test was used to compare continuous variables, such as age at diagnosis, between two groups. All P values reported were two-sided, and a P value of less than 0.05 was considered statistically significant. False discovery rates were calculated using the Benjamini-Hochberg procedure; FDR < 0.05 was used as the threshold for significance after correction for multiple hypothesis testing.

## Results

### Participant characteristics

The study included 1171 patients with qualified testing data (**Table 1**). These samples include all seven regions in China and can represent the characteristics of the Chinese ovarian cancer population. The age range of the enrolled patients was 12~86 years old (**Supplementary Figure S1**), the median age was 54 years old, and 247 patients had a family history of cancer. According to the statistics of clinical data, there was no significant correlation between family history of cancer and age of cancer onset. *BRCA-*positive patients were defined as those with likely pathogenic and/or pathogenic mutations in *BRCA1/2*, and 103 (8.8%) out of 1171 ovarian cancer patients were *BRCA*-positive.

**Table 1:** Participant characteristics of the cohort.

	With BRCA deleterious mutation (%)	Without BRCA deleterious mutation (%)	ALL
**Patients**	103	1068	1171
Family history			
**With**	16	231	247
**Without**	87	837	924
Age			
**<=47**	18	247	265
**>47**	85	821	906
**Average**	55.33	54.22	54.32
**Median**	56	54	54
**Range**	23~76	12~86	12~86
Region			
**East**	26	320	346
**North**	40	338	378
**NorthEast**	16	161	177
**NorthWest**	1	16	17
**Central**	3	33	36
**South**	8	77	85
**SouthWest**	9	123	132

### Characteristics of all mutations

Among the 1171 patients, a total of 19435 mutations were detected (**Supplementary Table S3**), with an average of more than 16 mutations per patient. These mutations contained 5657 types, of which 94.38% (5345) of which were SNPs, and the remaining 312 were INDELs. Categorized by mutation effect, the most common types were missense mutations (3105), followed by synonymous mutations (1704), 491 splice-site mutations, 177 frameshift mutations, 72 INDELs within coding frames, and 108 nonsense mutations. In the cohort, there were a total of 48 mutations present in 39 genes, each carried by more than 5% of the patients, and the five highest frequency mutations were in the following order: *MC1R*, c.359T>C; *ERCC5*, c.1586G>C; *PRSS1*, c.72C>T; *BARD1*, c.1075_1095del; and *KIT*, c.1638A>G. Meanwhile, we applied fit Chi-square calculation to evaluate these mutations with ovarian cancer risk and found MC1R:c.359T>C, ERCC5:c.1586G>C and PRSS1:c.72C>T that significantly fails to conform Hardy-Weinberg equilibrium (P-value<0.05). Notably, nearly 48% (23/48) of the high-frequency mutations were synonymous. We used the PATHVIEW software package (29) for the 39 genes and found that they were significantly enriched in DNA damage response repair pathways such as fanconi anemia pathway, homologous recombination, mismatch repair, nucleotide excision repair, and base excision repair (**Supplementary Figure S2A**).

**Figure 1:**
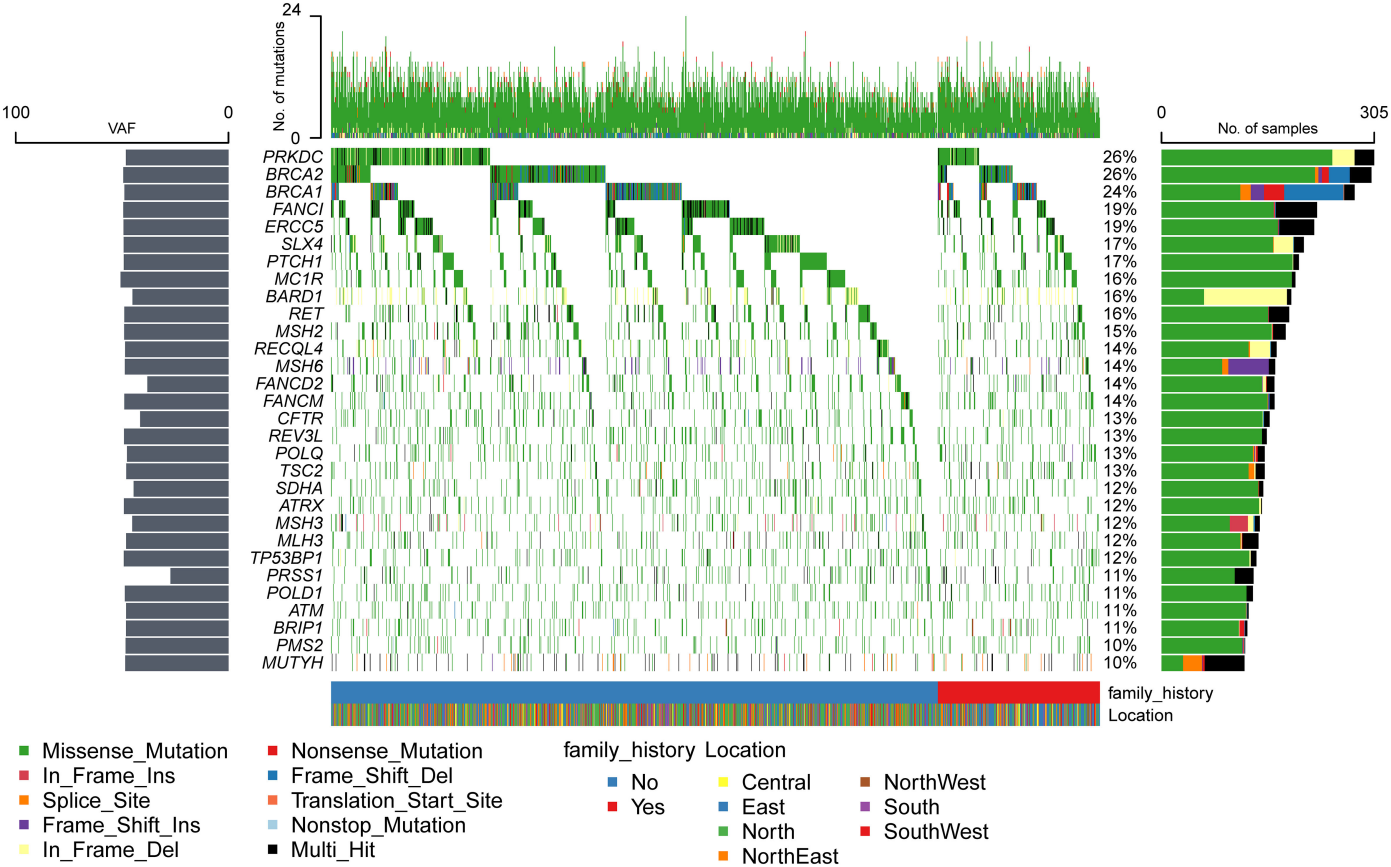
Mutation spectrum of high-frequency mutant genes.

**Figure 2:**
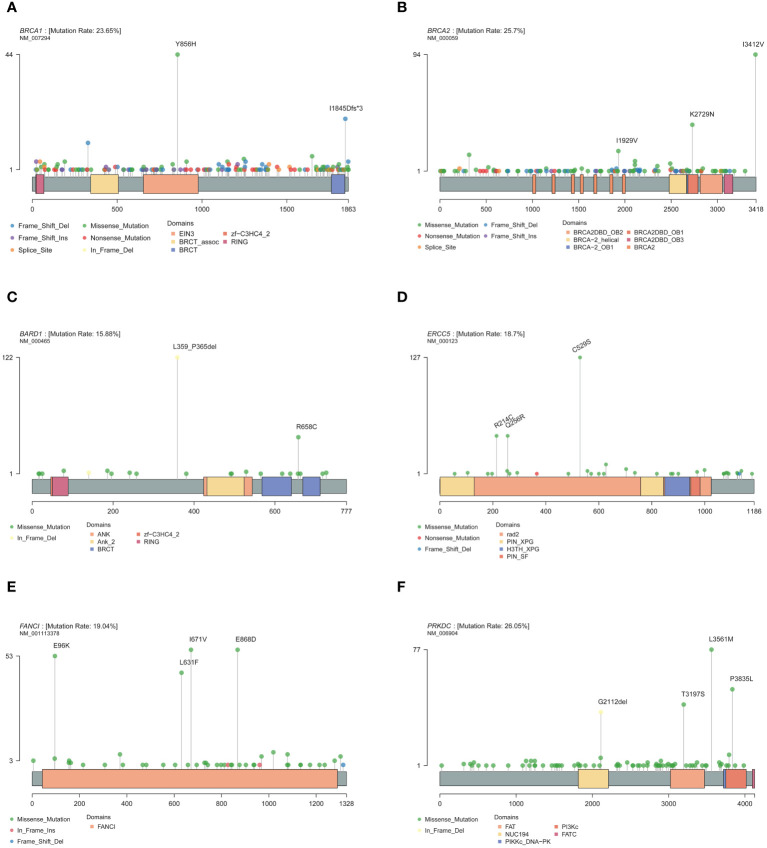
Mutation site of high-frequency mutant gene. **(A)** *BRCA1*. **(B)** *BRCA2*. **(C)** *BARD1*. **(D)** *ERCC5*. **(E)** *FANCI*. **(F)** *PRKDC*.

Mutations were detected in each of the 171 genes within the assay (**Supplementary Table S4**). The most common gene, *PRKDC*, was detected in 612 patients (approximately 52%), and the least common gene, *MAX*, was detected in only 1 patient. Genes with a high prevalence of mutations in more than 10% of the cohort were selected, and a total of 30 genes were obtained (**Figure 1**). The top 10 genes with high incidence were *PRKDC*, *BRCA2*, *BRCA1*, *FANCI*, *ERCC5*, *SLX4*, *PTCH1*, *MC1R*, *BARD1*, and *RET*. The 30 genes were significantly enriched in the fanconi anemia pathway (P value 2.55e-13, FDR 5.15e-11) and mismatch repair (P value 2.40e-10, FDR 2.42e-8) (**Supplementary Figure S2B**). An exploration of the distribution of mutation sites within genes showed single or multiple hotspot mutations in high-incidence mutated genes (**Figure 2**), such as *BRCA1*: c.2566T>C (p.Y856H), *BRCA2*: c.10234A>G (p.I3412V), *ERCC5*: c.1586G>C (p.C529S), *FANCI*: c.2011A>G (p.I671V), *PRKDC*: c.10681T>A (p.L3561M), and *BARD1*: c.1075_1095del (p.L359_P365del), suggesting that these sites were associated with ovarian cancer occurrence in China.

Compared with other high-frequency mutated genes (**Figure 1**), more INDEL mutations were detected in *BRCA1*, *BRCA2*, *PRKDC*, *BARD1*, *MSH6*, *RECQL4*, and *MSH3* genes, and more splice-site mutations were detected in the *BRCA1*, *MSH6*, *MUTYH*, and *TSC2* genes, which tended to cause greater functional changes. *BRCA1*, *BRCA2*, and *BARD1* are core genes of the homologous recombination repair pathway (30), and *MSH6* and *MSH3* are core genes of the mismatch repair pathway (31). The National Comprehensive Cancer Network (NCCN) guidelines (32) recommended screening for mutations in *RAD51D*, *EPCAM*, and *RAD51C*, which were detected in 5%, 4%, and 2% of the population, respectively. However, only the high-frequency mutated genes, *BRCA1* and *BRCA2*, were within the scope of NCCN-recommended screening. It implied that the mutated genes of ovarian cancer in the Chinese population differed significantly from those recommended by NCCN for ovarian cancer screening in both European and American populations.

### Characteristics of deleterious mutations

According to the classification of mutation function, a total of 954 (16.9%) were annotated as likely pathogenic or pathogenic mutations in 1041 patients (**Supplementary Table S3**). The top 10 genes with most deleterious mutations in the Chinese ovarian cancer population were *MC1R* (12%), *MLH1* (9%), *PRKDC* (8%), *KIF1B* (8%), *FANCM* (7%), *FANCI* (6%), *PRSS1* (6%), *SDHA* (6%), *BRCA2* (6%), and *CFTR* (5%) (**Supplementary Figure S3**). In addition, deleterious *BRCA1* mutations were detected with a frequency of about 3%, and *BRCA1* and *BRCA2* were detected in 103 patients (8.8%) patients. Pathway enrichment of these high-frequency genes showed that they were significantly enriched in the fanconi anemia pathway (P value 1.03e-06, FDR 2.00e-04). Analysis of the distribution of mutation sites within genes revealed the presence of single or multiple hotspot mutations in high-frequency mutation genes (**Supplementary Figure S4**), such as *MC1R*: c.359T>C (p.I120T), *MLH1*: c.1151T>A (p.V384D), *PRKDC*: c.10681T>A (p.L3561M), *ERCC5*: c.640C>T (p.R214C), *ERCC5*: c.767A>G (p.Q256R), *PTCH1*: c.2222C>T (p.A741V), and *RECQL4*: c.212A>G (p.E71G), suggesting that these sites were related to the occurrence and progression of ovarian cancer in China. The mutations observed were primarily missense mutations and were predominantly heterozygous, indicating that the dosage of the mutation had a significant impact on normal functional execution.

**Figure 3:**
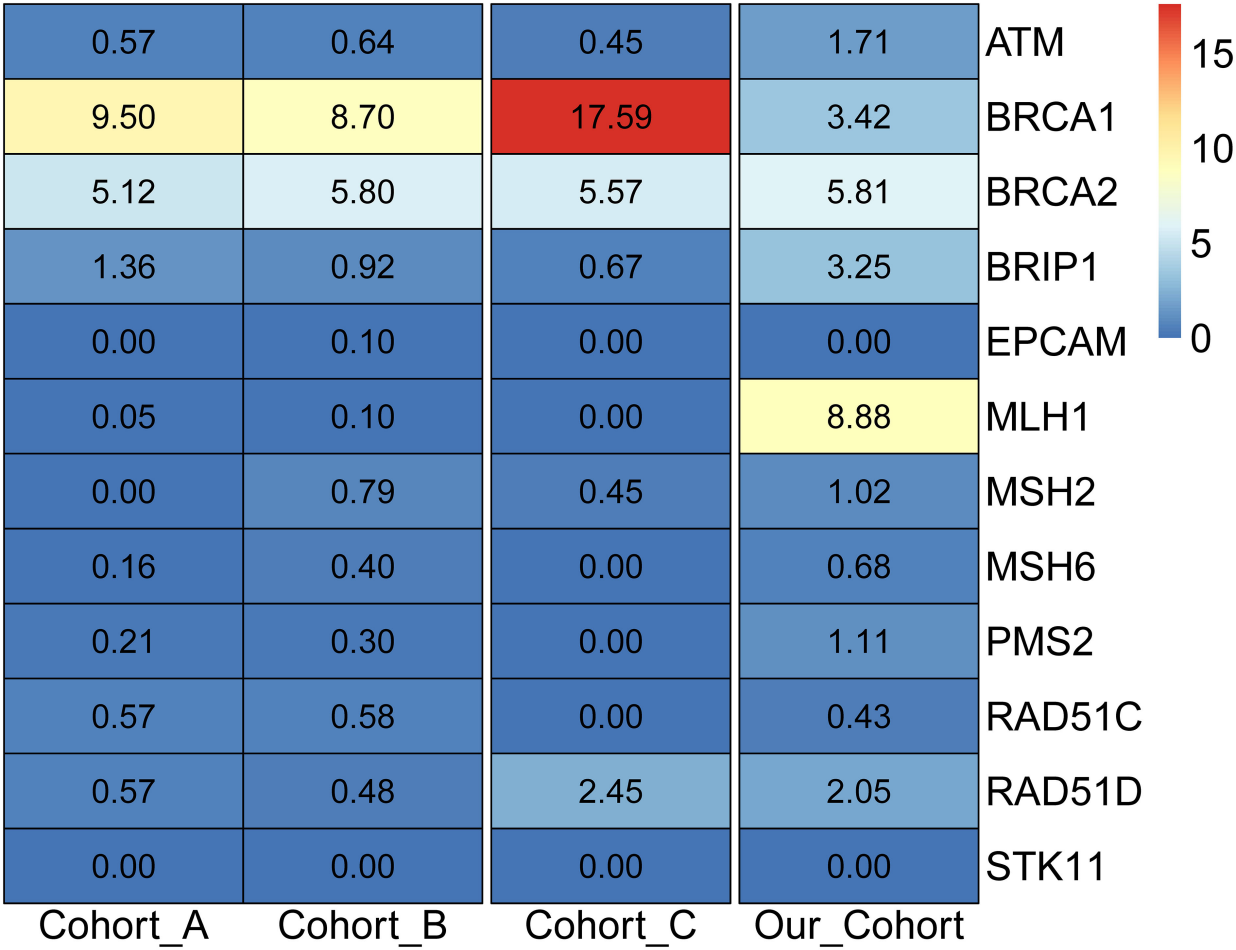
Detection rate of pathogenic mutations in NCCN ovarian cancer screening genes in different populations.

Considering only the NCCN-recommended ovarian cancer screening genes, it was noticed that no mutations were detected in the recommended *EPCAM* and *STK11* genes. Compared with the two American cohorts (cohorts A and B) (19, 33), and the four Chinese cohorts (cohorts C-F) (13, 18, 34, 35) (**Supplementary Table S5**), it was observed that the detection rates of the two cohorts in the United States were 18.10% and 18.81%, respectively. For the four cohorts in the Chinese population, the detection rate of Cohort C was 27.20%, and the other three cohorts were tested for only the *BRCA1* and *BRCA2* genes, with detection rates of 22.40%, 17.00%, and 28.40%, respectively. The detection rate of this study cohort was 28.35%, which was close to that of previous Chinese cohorts. Further analysis of the deleterious mutations in NCCN-recommended screening genes in foreign and domestic cohorts revealed that the *BRCA1* and *BRCA2* genes were generally detected at high frequencies in the American and Chinese populations (**Figure 3**), and the difference was that the MLH1 gene carried the highest frequency of deleterious mutations in this cohort. Meanwhile, it was discovered that all high-frequency mutation genes in the American population were within the screening scope recommended by the NCCN guidelines, while the high-frequency mutation genes in the Chinese cohort of ovarian cancer, such as *MC1R*, *PRKDC*, *KIF1B*, *FANCM*, *FANCI*, *PRSS1*, and *SDHA*, were not included in the scope of the NCCN guidelines, indicating that the NCCN guidelines based on the European and American populations were not suitable for ovarian cancer genetic screening in the Chinese population.

Among the 130 patients without detecting deleterious mutations, the high-frequency mutation genes were similar to those of all cohort populations, and pathway enrichment analysis showed that these genes were also significantly enriched in the fanconi anemia pathway (P value 1.60e-06, FDR 3.24e-04). Compared with the 1041 patients carrying deleterious mutations, there was no significant difference in age distribution (t-test P value 0.84) or family history of cancer (chi-square test P value 0.70). At the same time, it was identified that the genes with significantly high frequency in the group carrying deleterious mutations were mainly enriched in the homologous recombination (P value 1.02e-05, FDR 2.06e-03) and fanconi anemia pathway (P value 7.12e-05, FDR 7.20e-03).

### Characteristics of *BRCA1/2* gene mutations

A total of 190 *BRCA1* mutations and 169 *BRCA2* mutations were detected in the enrolled ovarian cancer population. Among the high-frequency *BRCA1* and *BRCA2* mutations, except for *BRCA1*:c.5470_5477delATTGGGCA (5.85% of all *BRCA1* mutations, 20/342), which was a pathogenic mutation, the others were all missense mutations annotated as benign. Further analysis revealed that *BRCA1*: c.2566T>C (p.Y856H) accounted for 12.87% of all *BRCA1* mutations (**Supplementary Figures S5A, B**), and *BRCA2*: c.8187G>T (p.K2729N) constituted 9.36% of all *BRCA2* mutations (**Supplementary Figures S5C, D**), were significantly higher in the East Asian population than in other populations, and were geographically characteristic *BRCA1/2* mutation in the Chinese ovarian cancer population. However, *BRCA2*:c.10234A>G (p. I3412V) comprised 23.15% of all *BRCA2* mutations (**Supplementary Figures S5E, F**), with a frequency of 2.40% in East Asian populations and 8.03% in this ovarian cancer cohort. Notably, its prevalence exceeds 10% in normal populations in the Americas and Africa, displaying that it was not a geographically characteristic mutation in the Chinese population.

Co-mutation in *BRCA1* and *BRCA2* occurred in 99 patients, representing 8.5% (99/1171) of the entire cohort. Analysis of the characteristics of the *BRCA1* and *BRCA2* co-mutation group and other patients, it was observed that there was no statistical difference in family history distribution (chi-square test, P value 0.68). However, in terms of age (**Supplementary Figure S6**), the group carrying *BRCA1* and *BRCA2* co-mutations had a significantly earlier age of cancer onset than the rest patients (mean age, 51 VS 54.5 years, Wilcoxon test P value 7.30e-03). Similar results have been reported in multiple studies (34, 36), implying that *BRCA1* and *BRCA2* were associated with the early onset of cancer.

Comparative analysis of the difference in ovarian cancer mutation genes between the *BRCA1/2* mutation group and the non-mutation group uncovered that *FAM175A*, *EMSY*, *PTCH1*, and *HNF1B* were significantly highly mutated in the group without *BRCA1/2* mutations (**Supplementary Figure S7A**), particularly *PTCH1*: c.3907C>T (p.R1303C) (**Supplementary Figure S7B**). When considering only the difference in deleterious mutations between the two groups, it was found that *FAM175A* was equally significant in the group without *BRCA1/2* mutations (**Supplementary Figure S7C**).

### Mutations associated with family history

**Figure 4:**
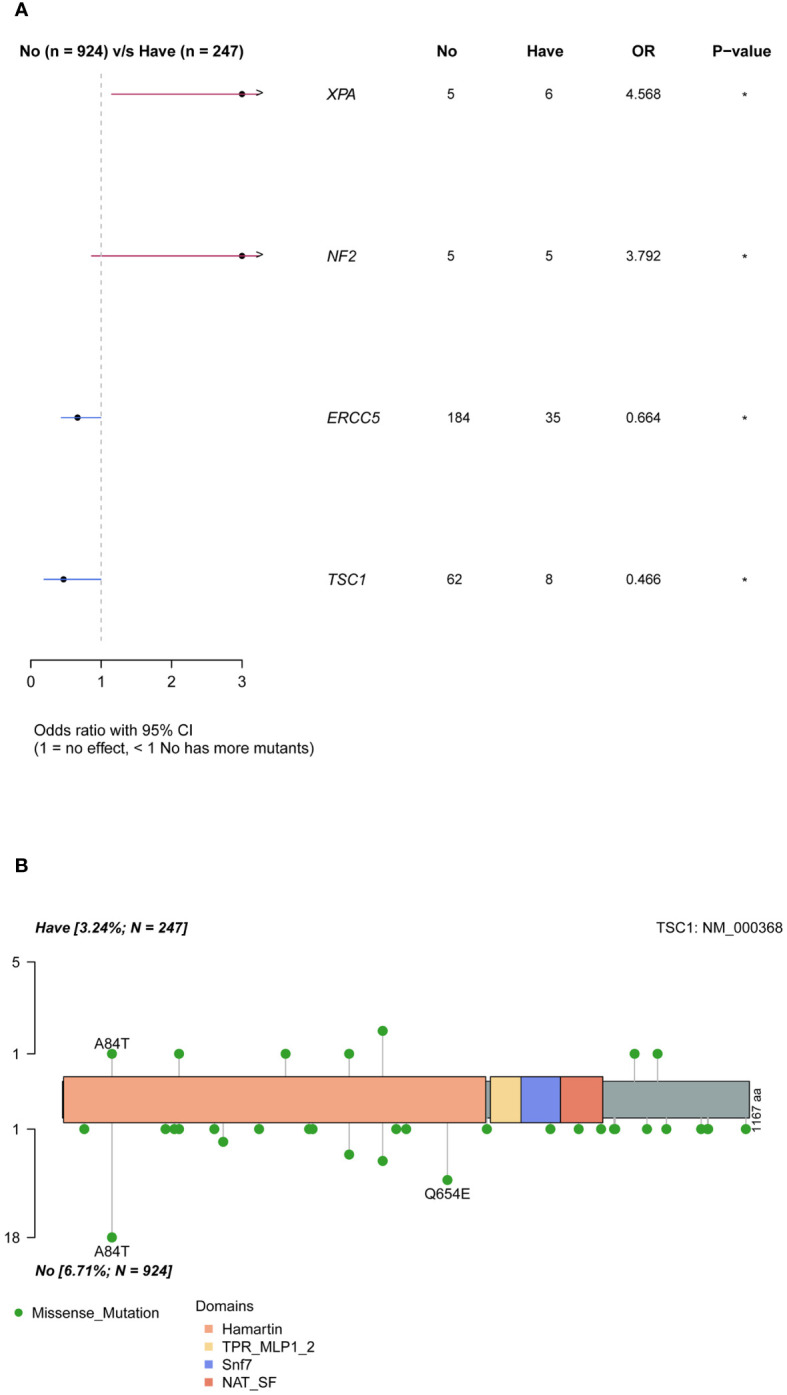
Differences of mutated genes in groups with and without cancer family history. **(A)** all mutations. **(B)** difference of *TSC1* mutation sites in groups with and without cancer family history.

Ovarian cancer exhibited a notable pattern of family inheritance and aggregation, primarily attributed to the transmission of tumor-associated germline mutations to the offspring along with the reproductive process. The enrollment cohort consisted of 247 samples with a family history of cancer and 924 samples without. By comparing the differences in mutation genes between the two groups (**Figure 4A**), it was observed that the *XPA* and *NF2* genes were significantly more frequently mutated in the group with a family history of cancer (P <0.05), while the *ERCC5* and *TSC1* genes were more common in the group without a family history of cancer (P <0.05). Analysis of the mutation sites in these genes revealed that the *TSC1* gene had a significantly higher frequency of mutation c.250G>A (p.A84T) in the population without a family history (**Figure 4B**).

### Mutations associated with age of diagnosis

The analysis of hereditary mutations in each sample revealed a decrease in the total number of mutations as onset age increased within the patient population (**Supplementary Figure S8A**), suggesting that the occurrence of ovarian cancer in the younger age group was mainly related to genetic factors. According to the age distribution of the cohort, the patients in the first quartile of the age range (47 years and younger) were categorized into the younger age group and were compared with the group older than 47 years. Fisher’s exact test was performed on the mutations of each gene in the two age groups (**Supplementary Figure S8B**). The results showed that *ERCC4*, *CHEK2*, and *PDGFRA* were significantly more mutated in the younger group (P value<0.05), while *POLH* was significantly more common in the older group.

### Mutations associated with native place

**Figure 5:**
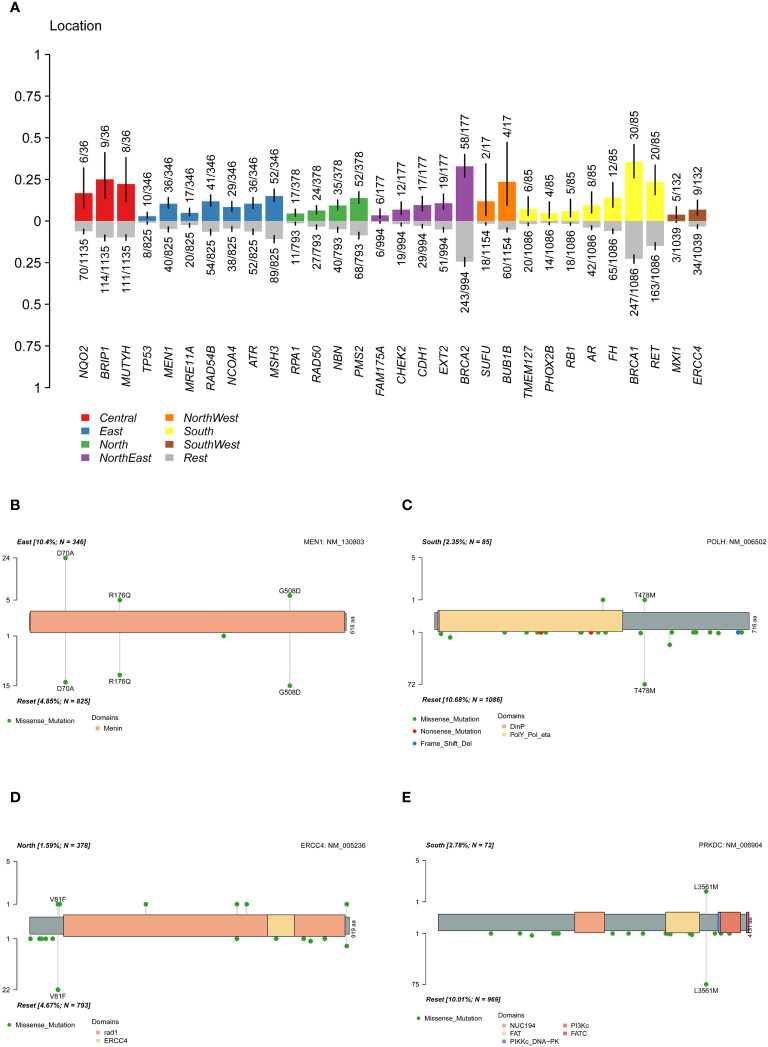
Differences of mutation genes among different location. **(A)** difference of mutated gene between a single region and other regions. **(B)** difference of *MEN1* mutation sites between the East China and the reset. **(C)** difference of *POLH* mutation sites between the South China and the reset. **(D)** difference of *ERCC4* mutation sites between the North China and the reset. **(E)** difference of *PRKDC* deleterious mutation sites between the South China and the reset.

Regional characteristics of cancer-related mutation genes were highly prevalent due to differences in the ancestral genetic backgrounds of various regions. In the cohort, a Fisher’s exact test was conducted on each mutated gene between a single region and other regions (**Figure 5A**). The results showed that *MEN1* was significantly more highly mutated in the East China group, *MXI1* gene in the Southwest China group, *TMEM127* and *FH* in the South China population, *RPA1* in the North China population, and *CDH1*, *CHEK2*, *FAM175A*, and *EXT2* in the Northeast population. *ERCC4* and *HMMR* were significantly more common in the non-North China population, while *POLH* was more common in the non-South China population. The pathway enrichment revealed that high-frequency mutated genes in North China were significantly enriched in the fanconi anemia pathway (p-value 6.20e-07, FDR 1.25e-04), homologous recombination (p-value 7.15e-07, FDR 7.22e-04), nucleotide excision repair (P value 3.49e-05, FDR 2.35e-03), mismatch repair (P value 4.82e-04, FDR 0.02), while in South China, in the fanconi anemia pathway (P value 2.12e-05, FDR 4.28e-03). When only deleterious mutations were considered (**Supplementary Figure S9**), *CDH1* and *FAM175A* were significantly more frequently mutated in the Northeastern population, while *ERCC4* was in non-North China populations, and the *PRKDC* in non-South China populations. The results indicated that differences in the geographic scope of China also led to differences in cancer susceptibility genes for ovarian cancer.

By analyzing the mutation carrier rates of the differential genes, we found that *MEN1*: c.209A>C (p.D70A) was highly prevalent in the East China population (**Figure 5B**), while *MEN1*: c.1523G>A (p.G508D) and c.527G>A (p.R176Q) in the populations of other regions. Furthermore, a significant high-frequency mutation, *POLH*: c.1433C>T (p.T478M) (**Figure 5C**), was observed in non-South China populations and *ERCC4*: c.241G>T (p.V81F) in non-North China populations (**Figure 5D**). The pathogenic *PRKDC*: c.10681T>A (p.L3561M) mutation was significantly more common in the non-South China population (**Figure 5E**). The presence of highly mutated genes in different regions suggested that precise treatment of ovarian cancer in the Chinese population should be based on characteristic mutation data specific to this population.

## Discussion

By gathering germline mutation data from 1171 ovarian cancer samples across China, a mutation database of susceptibility genes was constructed based on the genetic background of the Chinese population. Our findings revealed that high-frequency mutated genes had hotspot mutations. In order of prevalence, mutated genes with a frequency of more than 16% in the enrolled ovarian cancer cohort population were, *PRKDC*, *BRCA2*, *BRCA1*, *FANCI*, *ERCC5*, *SLX4*, *PTCH1*, *MC1R*, *BARD1*, and *RET*. Compared with the NCCN guidelines on ovarian cancer screening genes based on the genetic characteristics of the European and American populations, we observed that the guideline-recommended genes *RAD51D*, *EPCAM*, and *RAD51C* were only detected in less than 5% of the population, while genes with high-frequency mutations in the Chinese population, such as *PRKDC*, *FANCI*, and *ERCC5*, were not included in the screening scope. Considering only deleterious mutations, it showed that the carrying rates of deleterious mutations of the ovarian cancer screening genes recommended by the NCCN guidelines also varied significantly across different ethnic groups. Furthermore, the high-frequency mutated genes in the American population fell within the screening range recommended by the NCCN guidelines, while the high-frequency mutated genes, *MC1R*, *PRKDC*, and *KIF1B* in the Chinese ovarian cancer cohort were not covered. The results highlighted that the mutation characteristics of the Chinese ovarian cancer population were significantly different from those of the European and American populations. Therefore, it was imperative to formulate genetic screening guidelines for ovarian cancer that aligned with the genetic characteristics of the Chinese population.

*BRCA1* deleterious mutations were detected in 40 samples (3.42%) and *BRCA2* deleterious mutations in 68 samples (5.81%). In previous studies of ovarian cancer in China, the carrying rates of *BRCA1* were generally higher than our results. Three Chinese population cohort studies (13, 34, 35) showed that the carrier frequencies of *BRCA1* ranged from 13.1% to 20.8% and those of *BRCA2* were between 3.9% and 7.6%. In two studies of the American population (19, 37), the frequencies of *BRCA1* were 8.6% and 9.5%, respectively, while the frequencies of *BRCA2* were 5.2% and 5.1%. The low *BRCA1* positive rate in our cohort was attributed to the geographical differences in the enrollment cohort and the high-grade serous carcinoma in the other study population, suggesting that the deleterious mutations of *BRCA1* were associated with a greater incidence of malignant ovarian cancer.

The prevalence of mutations at different sites in genes varied greatly among different populations. Three geographically characteristic mutations were identified in this study: *BRCA1*: c.5533_5540del (p.I1845Dfs*3), which was detected in 38 samples in this cohort (1.69%), has been identified as a founder mutation in four studies of Chinese ovarian cancer populations (13, 14, 34, 35), and was confirmed to have appeared in the Han Dynasty of China 2000 years ago (36); *BRCA1*: c.2566T>C (p.Y856H), which was detected in 93 (4.13%) samples in the current cohort, and was also identified as the founder mutation in one Chinese population study of ovarian cancer (14); and *BRCA2*: c.8187G>T (p.K2729N), which was detected in 74 (3.28%) samples in the current cohort, with no similar reports in China. Whether this mutation is a founder mutation requires further research. Among the founder mutations identified in previous studies of ovarian cancer in the Chinese population, except for c.1081delG, c.2612C>T, c.3548A>G, c.4837A>G, and c.5154G>A in the *BRCA1*, and c.3337C>T in the *BRCA2*, the rest of the mutations were detected in a few samples, and could not be presumed to be geographically specific mutations. Possible reasons for this include small enrollment cohorts in other studies and samples from a particular region or a particular subtype of cancer. Moreover, founder mutations identified in other ethnic groups (38, 39), such as Europeans and Americans, were not detected at high frequencies in this cohort, indicating that there were obvious differences in the genetic backgrounds of different ethnic groups.

​Ovarian cancer had the characteristics of high familial incidence, and the results of our cohort showed that *XPA* and *NF2* were associated with familial inheritance of ovarian cancer. *XPA* is a core gene for nucleic acid excision repair and has been extensively studied (40, 41). The *NF2* is a tumor suppressor gene, and the encoded protein is a linker protein between cytoskeletal components and proteins in the cell membrane, which is involved in regulating contact-dependent inhibition of cell proliferation and plays a key role in intercellular adhesion and transmembrane signaling (42). Mutations in the *NF2* are associated with tumorigenesis and metastasis (43). Previous studies have focused on somatic mutations in *NF2* and found that these mutations are highly prevalent mainly in brain tissue (44), with a frequency of only 1% in the ovarian cancer population. Another study (45) revealed that the frequency of *NF2* detection in the ovarian cancer population was 2.56%, which is close to the germline carrier frequency in the current cohort (2.31%). The germline mutation of *NF2* in ovarian cancer has rarely been studied, and the relationship between germline mutations in *NF2* and familial inheritance of ovarian cancer requires further investigation.

Age is a crucial factor influencing the occurrence of cancer. As age increased, the number of germline mutations tended to decrease, but there was a noticeable difference in germline mutation genes between younger and older cancer patients. We identified significant high-frequency mutations in the *ERCC4*, *CHEK2*, and *PDGFRA* genes in young patients with ovarian cancer. The *CHEK2* gene is involved in cell cycle checkpoint regulation and is a tumor suppressor gene (46). The gene is activated by DNA damage and encodes a protein that inhibits the CDC25C phosphatase, blocking entry into the mitotic phase and resulting in the cell cycle arrest in the G1 phase. In addition, the protein interacts with and phosphorylates the BRCA1 protein, causing its activation after DNA damage. However, there are few studies on the association of *CHEK2* mutations and age at the onset of ovarian cancer. A study (47) in Poland containing 2012 ovarian cancer patients showed that the average age of the ovarian cancer group with *CHEK2* was 38 years, while the average age of the *CHEK2*-negative ovarian cancer group was 49 years.

In conclusion, this study constructed a comprehensive and accurate susceptibility gene dataset of ovarian cancer in the Chinese population and provided abundant data for the formulation of genetic screening and treatment guidelines for ovarian cancer tailored to the population. Moreover, the Chinese ovarian cancer population had characteristic high-frequency mutated genes and hotspots. Additionally, this study was the first to conduct an association analysis of patient characteristics across 171 genes, including DDR pathway-related genes, and identified characteristic mutated genes and sites linked to age, family history of cancer, and specific geographic regions. For further research, on the one hand, it is necessary to combine comprehensive clinical records and multi-omics information to accurately identify mutations associated with ovarian cancer. On the other hand, additional functional validation studies are needed to confirm the detailed mechanism of susceptibility genes and cancer occurrence and to provide data support for medical policy formulation, drug development, and clinical patient treatment.

## Data availability statement

The original contributions presented in the study are included in the article/**Supplementary Material**. Further inquiries can be directed to the corresponding authors.

## Ethics statement

The studies involving humans were approved by Medical Ethics Committee of West China Second University Hospital. The studies were conducted in accordance with the local legislation and institutional requirements. Written informed consent for participation in this study was provided by the participants’ legal guardians/next of kin.

## Author contributions

JW: Writing – original draft, Visualization, Software, Methodology, Investigation, Formal analysis, Data curation. KF: Writing – original draft, Data curation. MZ: Writing – original draft, Data curation. LL: Writing – original draft, Formal analysis. MN: Writing – review & editing, Formal analysis. HS: Writing – review & editing. RY: Writing – review & editing, Funding acquisition, Conceptualization. MT: Writing – review & editing, Conceptualization.

## Supplementary material

Supplementary Material 1 Mutation detection methods.

Supplementary Figure 2 Pathway enrichment results. **(A)** the 39 genes with high frequency mutations. **(B)** the 30 most common mutated genes.

Supplementary Figure 4 Mutation site of genes with high-frequency deleterious mutation. **(A)** *MC1R*. **(B)** *MLH1*. **(C)** *PRKDC*. **(D)** *ERCC5*. **(E)** *PTCH1*. **(F)** *RECQL4.*

Supplementary Figure 5 Comparison of *BRCA1* and *BRCA2* high-frequency mutations in the enrollment cohort and the detection frequency of GnomAD database. **(A)** Frequency of *BRCA1* c.2566T>C in different population. **(B)** Frequency of *BRCA1* c.2566T>C in Chinese ovarian cancer patients and normal people. **(C)** Frequency of *BRCA2* c.8187G>T in different population. **(D)** Frequency of *BRCA2* c.8187G>T in Chinese ovarian cancer patients and normal people. **(E)** Frequency of *BRCA2* c.10234A>G in different population. **(F)** Frequency of *BRCA2* c.10234A>G in Chinese ovarian cancer patients and normal people. AFR, African/African American; AMR, Latino/Admixed American; ASJ, Ashkenazi Jewish; EAS, East Asian; FIN, European (Finnish); NFE, European (non-Finnish); OTH, Other.

Supplementary Figure 7 Mutation gene differences between *BRCA* and non-*BRCA* groups. **(A)** all mutations. **(B)** difference of *PTCH1* mutation sites between *BRCA* and non-*BRCA* groups. **(C)** deleterious mutations.

Supplementary Figure 8 Differences in mutation genes among the different age. **(A)** The distribution of number of sample mutations at different ages. **(B)** Differences in mutation genes between the younger group and the older group.
