# Supplementary material for: Mutation characteristics of cancer susceptibility genes in Chinese ovarian cancer patients
Source: Front Oncol. 2024 May 16;14:1395818. doi: 10.3389/fonc.2024.1395818 (PMC11137316; doi:10.3389/fonc.2024.1395818)

Figure S6. Differences in age distribution between BRCA1 and BRCA2 co-mutation group and other patients

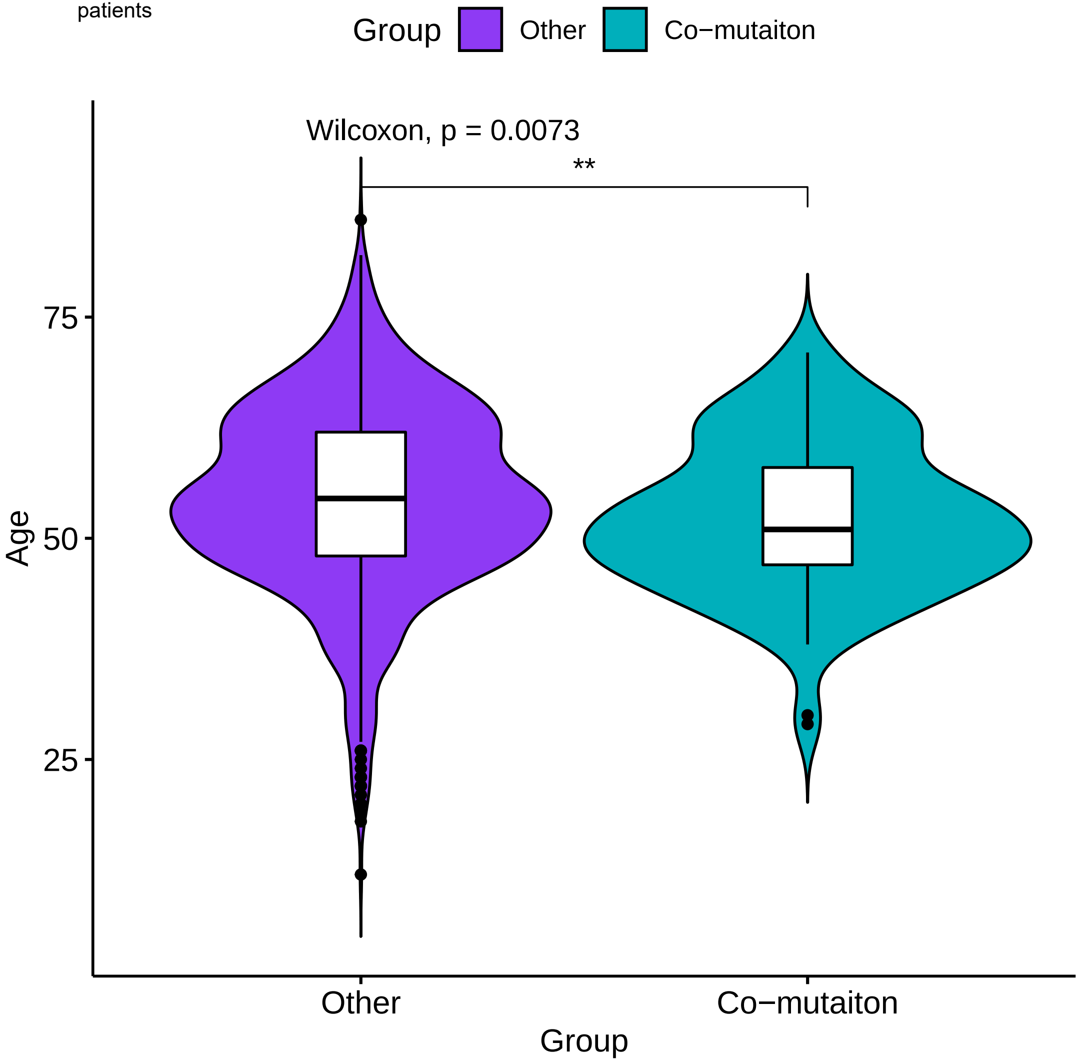

Supplement: Supplementary file 1 [file Image_6.pdf]
