# Supplementary figures and images for: Mutation characteristics of cancer susceptibility genes in Chinese ovarian cancer patients
Source: Front Oncol. 2024 May 16;14:1395818. doi: 10.3389/fonc.2024.1395818 (PMC11137316; doi:10.3389/fonc.2024.1395818)

Figure S7. Mutation gene differences between BRCA and non-BRCA groups

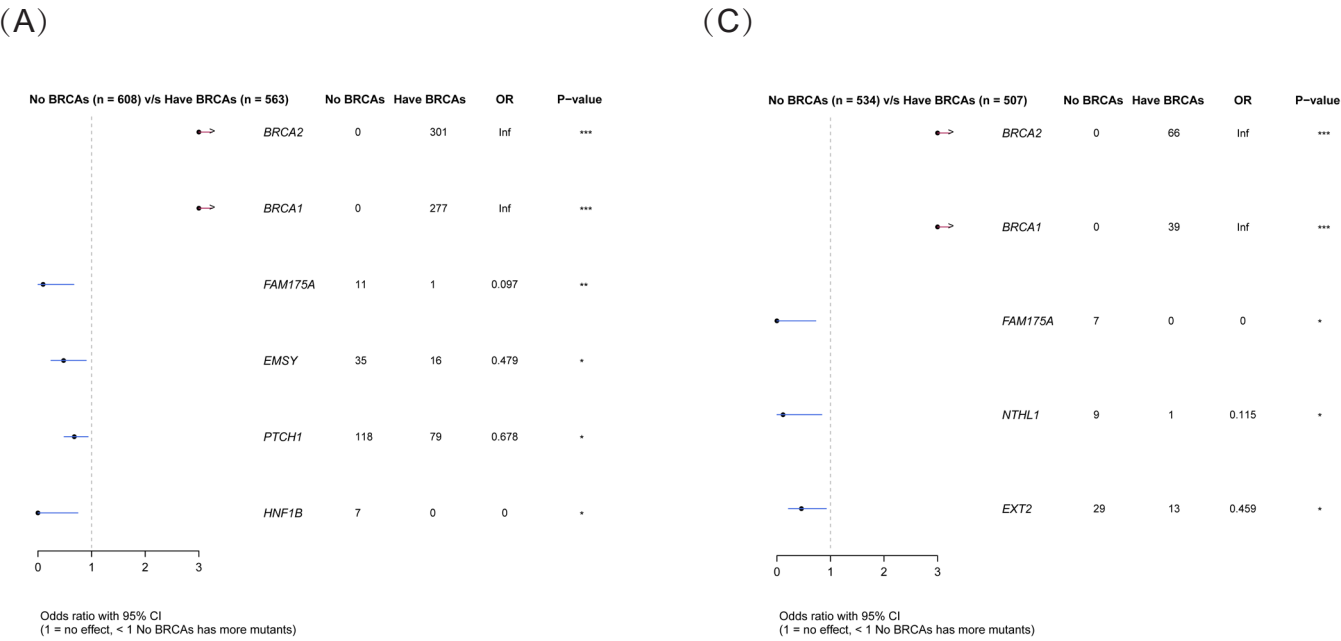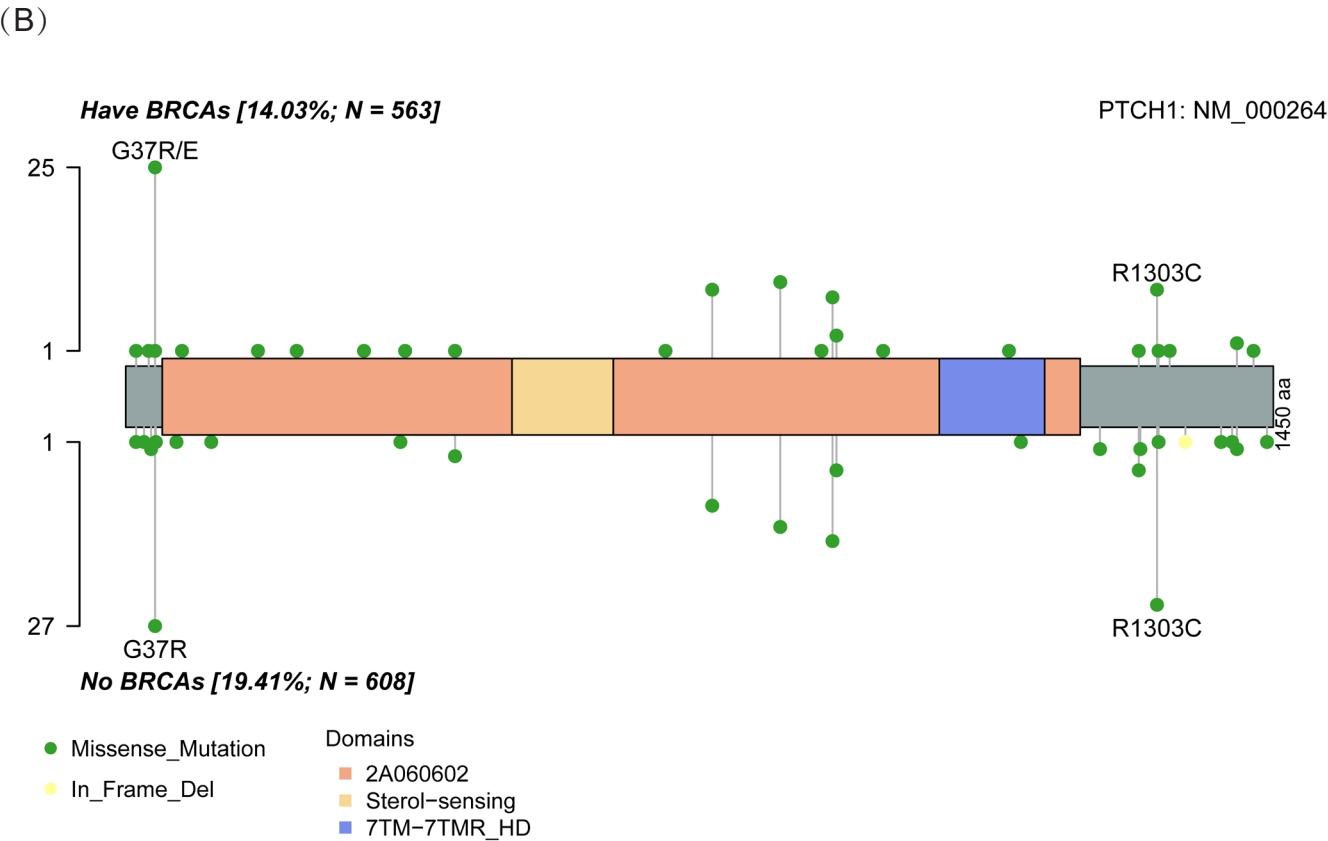

Supplement: Supplementary file 2 [file Image_7.pdf]

Figure S8. Differences in mutation genes among the different age

(A)

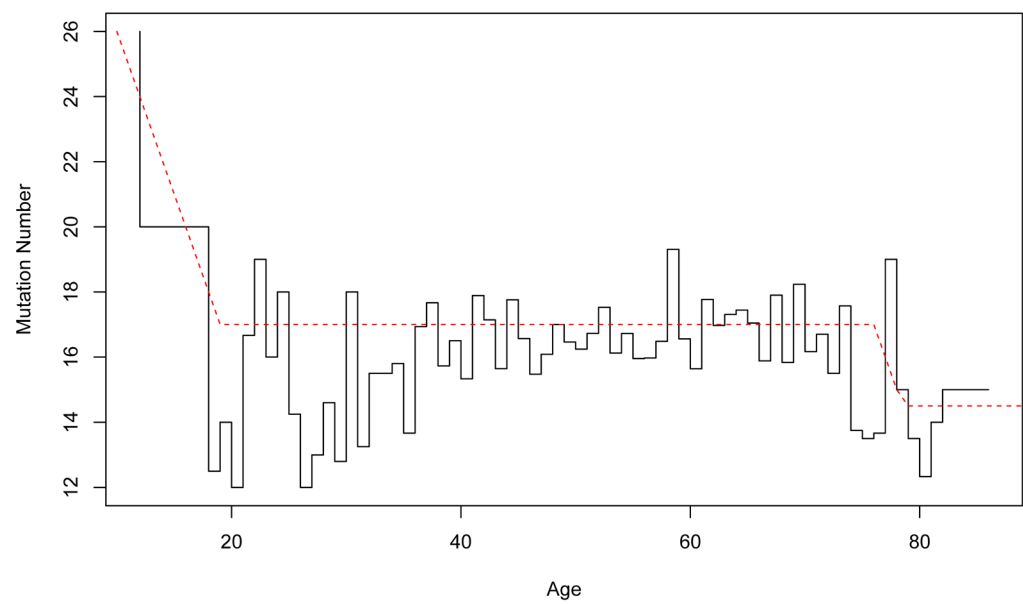

(B)

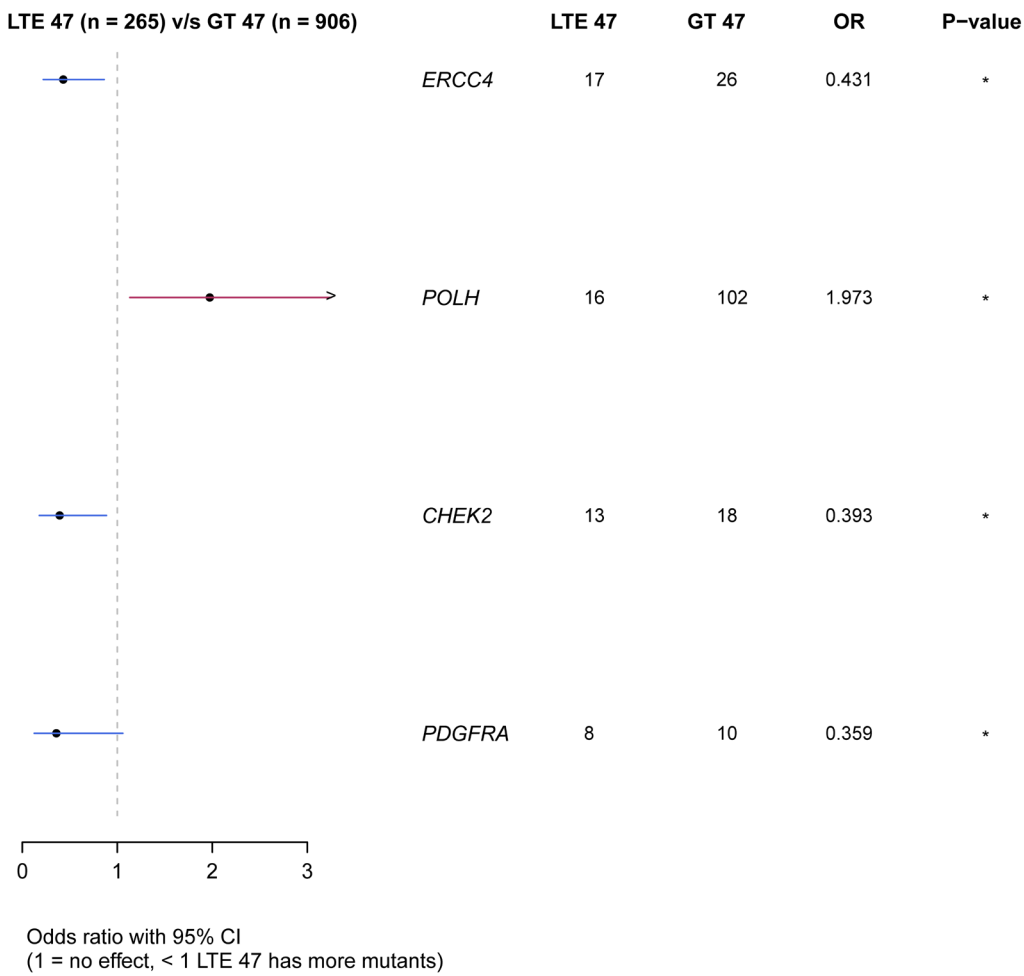

Supplement: Supplementary file 3 [file Image_8.pdf]

Figure S9 Differences of deleterious mutation genes among different location

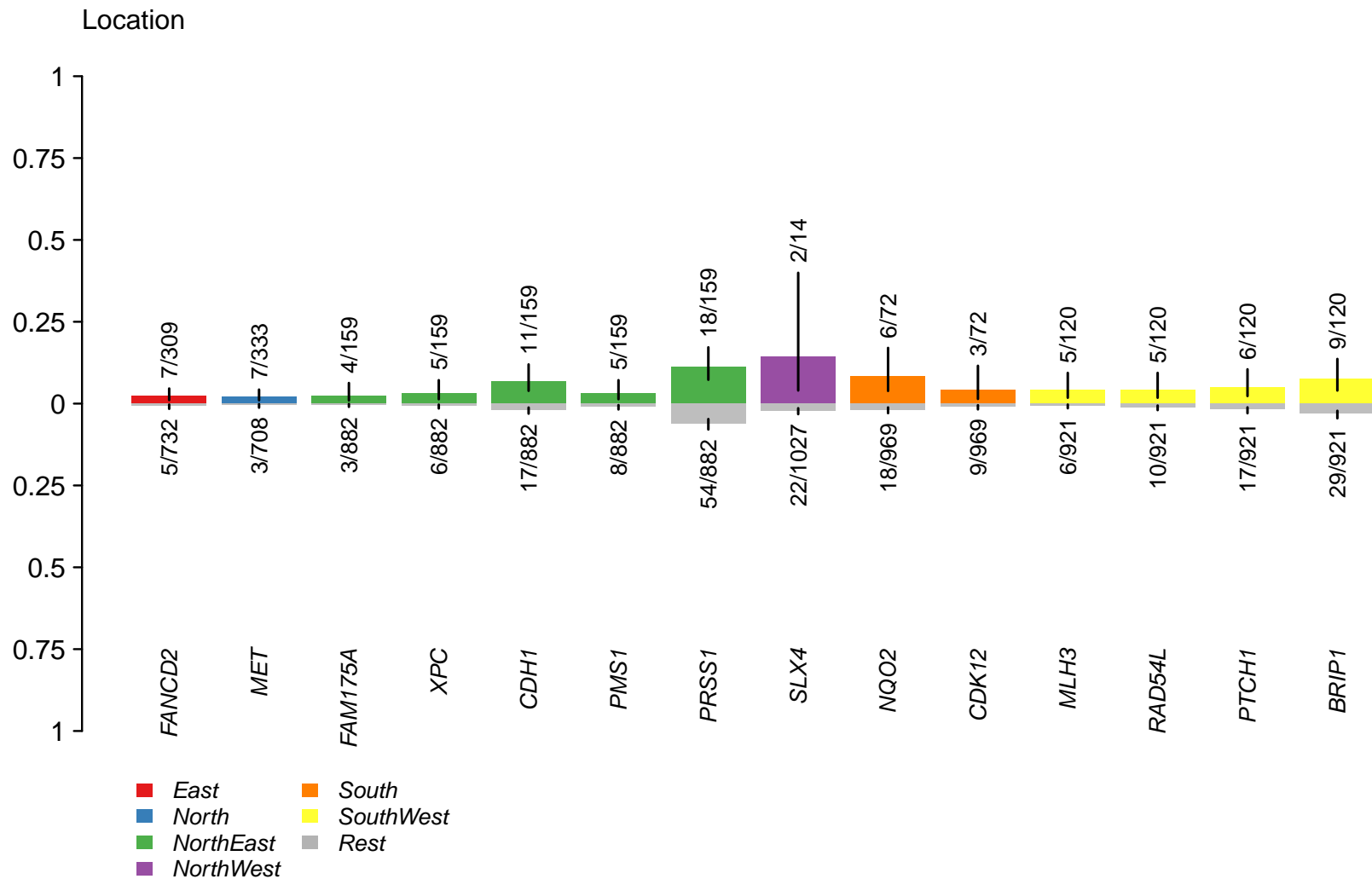

Supplement: Supplementary file 4 [file Image_9.pdf]

Figure S1. Age distribution of the cohort

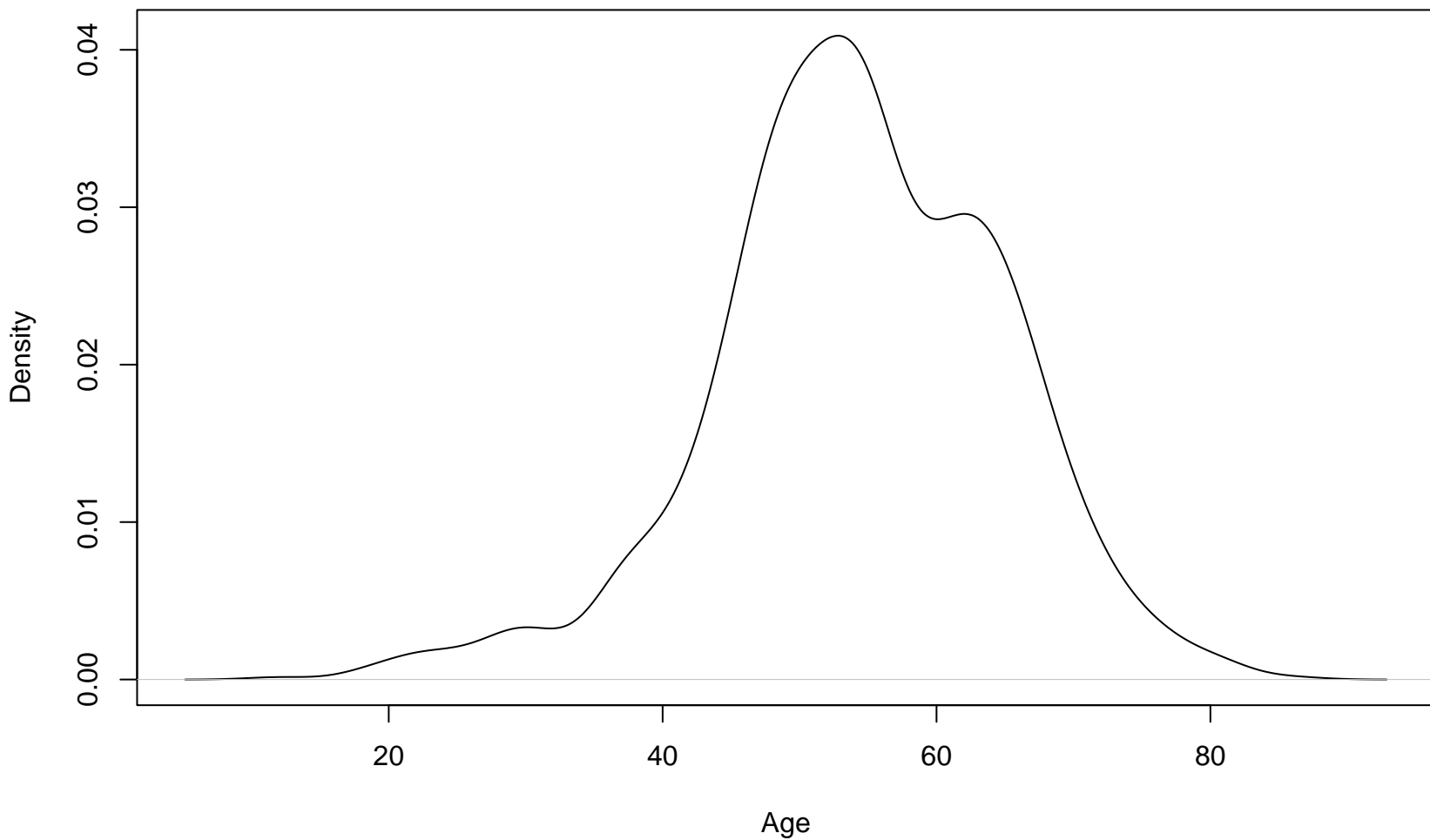

Supplement: Supplementary Figure 2 — Pathway enrichment results. (A) the 39 genes with high frequency mutations. (B) the 30 most common mutated genes. [file Image_1.pdf]

Figure S2. Pathway enrichment results

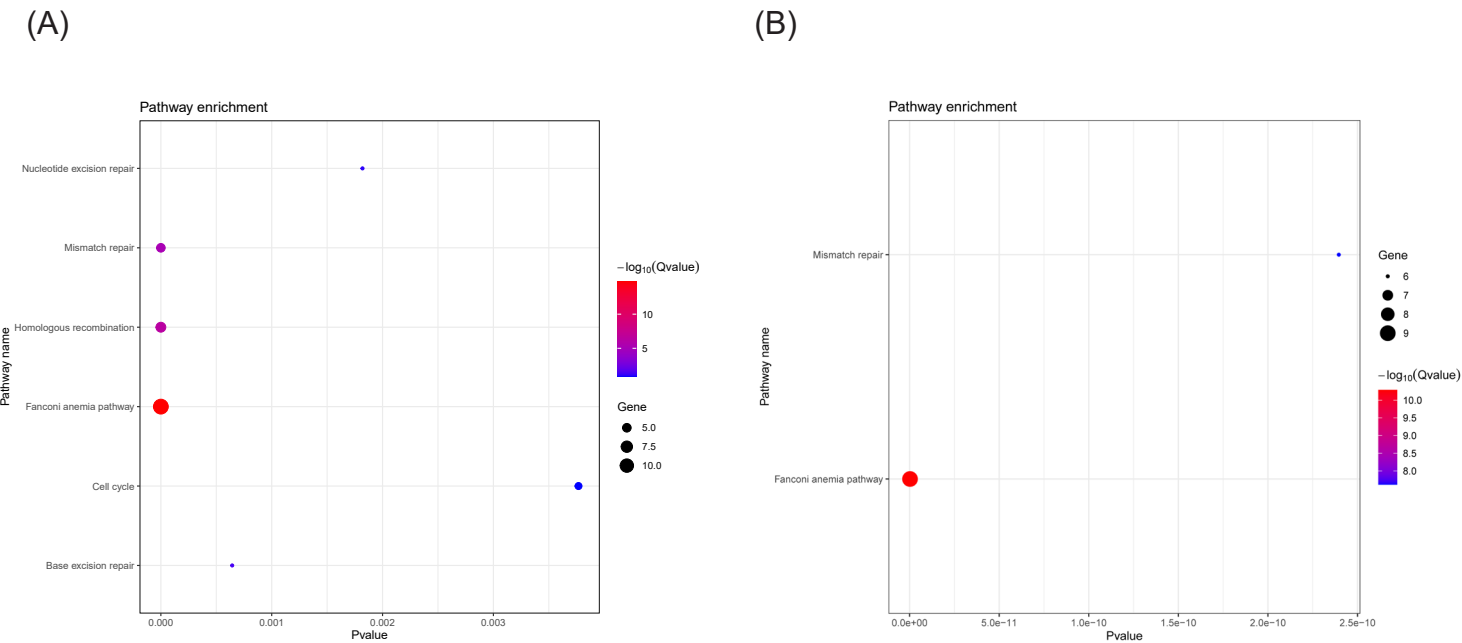

Supplement: Supplementary Figure 4 — Mutation site of genes with high-frequency deleterious mutation. (A) MC1R. (B) MLH1. (C) PRKDC. (D) ERCC5. (E) PTCH1. (F) RECQL4. [file Image_2.pdf]

Figure S3. Mutations spectrum of genes with high-frequency deleterious mutation

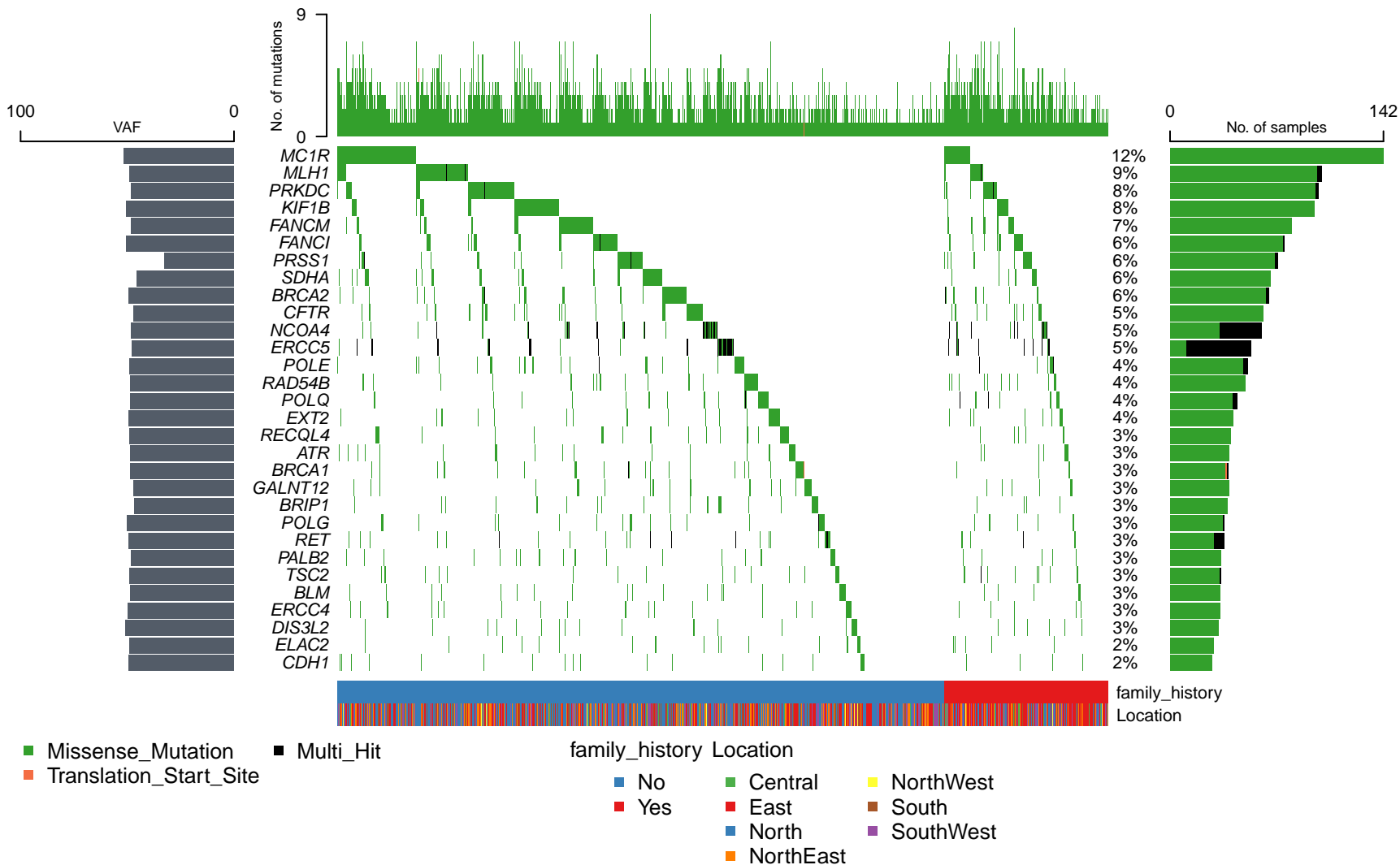

Supplement: Supplementary Figure 5 — Comparison of BRCA1 and BRCA2 high-frequency mutations in the enrollment cohort and the detection frequency of GnomAD database. (A) Frequency of BRCA1 c.2566T>C in different population. (B) Frequency of BRCA1 c.2566T>C in Chinese ovarian cancer patients and normal people. (C) Frequency of BRCA2 c.8187G>T in different population. (D) Frequency of BRCA2 c.8187G>T in Chinese ovarian cancer patients and normal people. (E) Frequency of BRCA2 c.10234A>G in different population. (F) Frequency of BRCA2 c.10234A>G in Chinese ovarian cancer patients and normal people. AFR, African/African American; AMR, Latino/Admixed American; ASJ, Ashkenazi Jewish; EAS, East Asian; FIN, European (Finnish); NFE, European (non-Finnish); OTH, Other. [file Image_3.pdf]

Figure S4. Mutation site of genes with high-frequency deleterious mutation

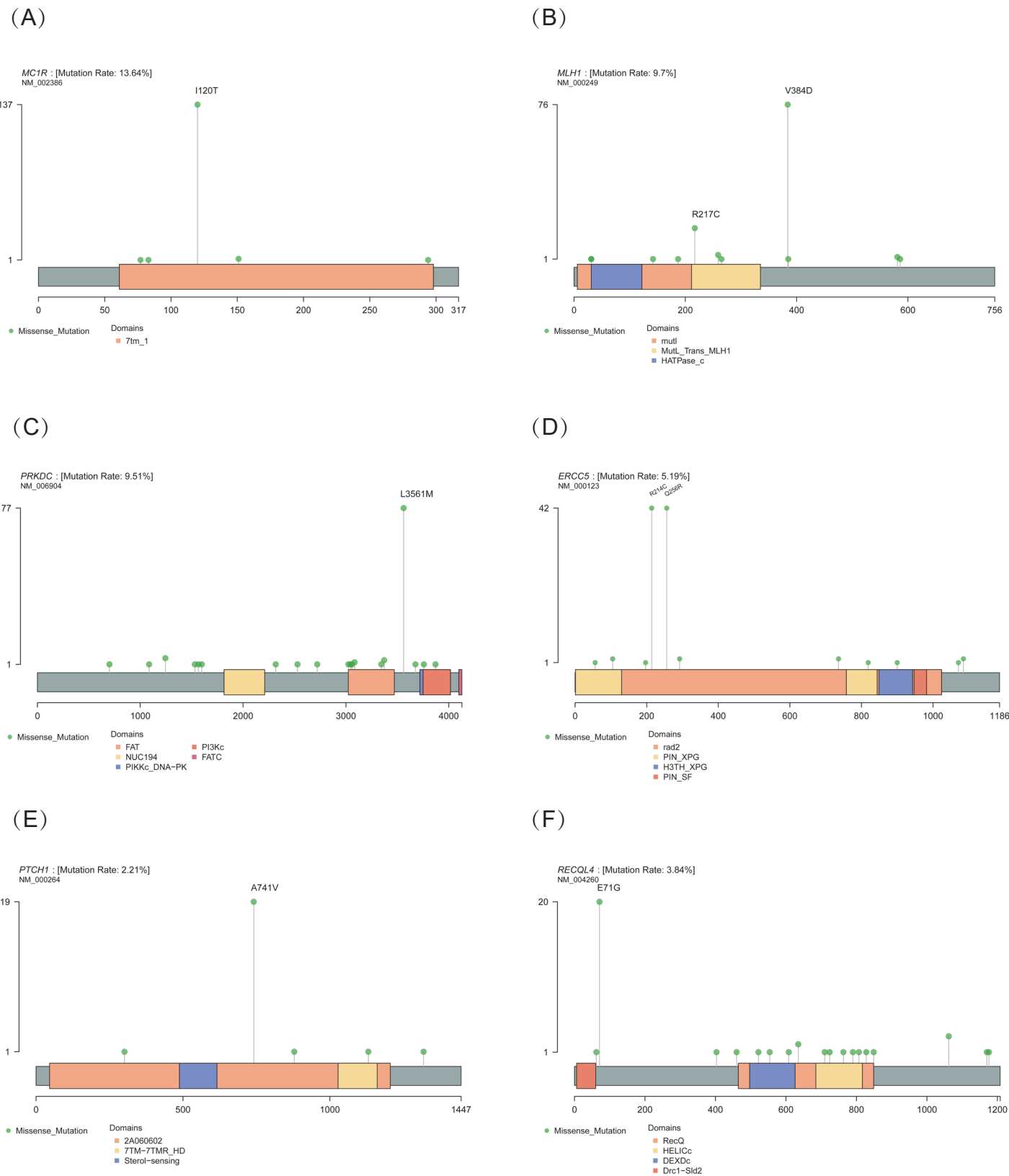

Supplement: Supplementary Figure 7 — Mutation gene differences between BRCA and non-BRCA groups. (A) all mutations. (B) difference of PTCH1 mutation sites between BRCA and non-BRCA groups. (C) deleterious mutations. [file Image_4.pdf]
