# Supplementary material for: Mutation characteristics of cancer susceptibility genes in Chinese ovarian cancer patients
Source: Front Oncol. 2024 May 16;14:1395818. doi: 10.3389/fonc.2024.1395818 (PMC11137316; doi:10.3389/fonc.2024.1395818)

The Chinese ovarian cancer population had characteristic high-frequency mutated genes and hotspots

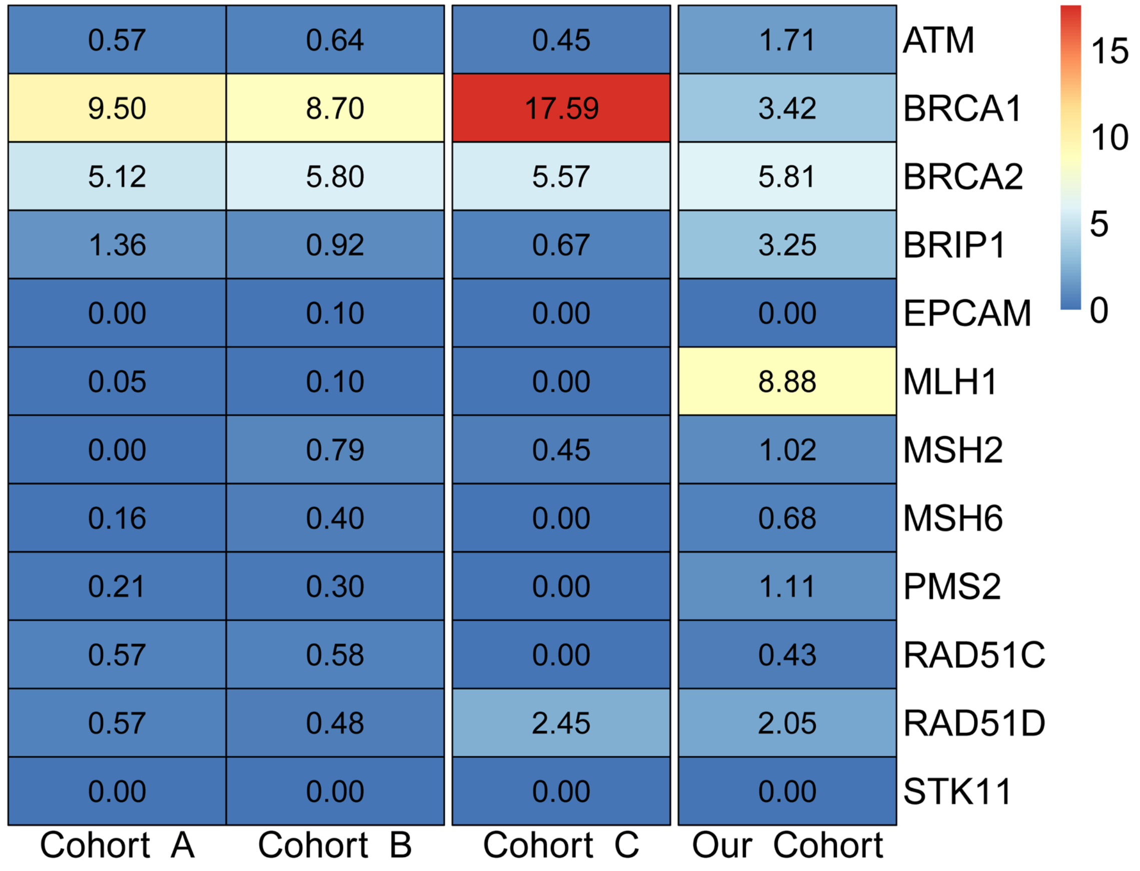

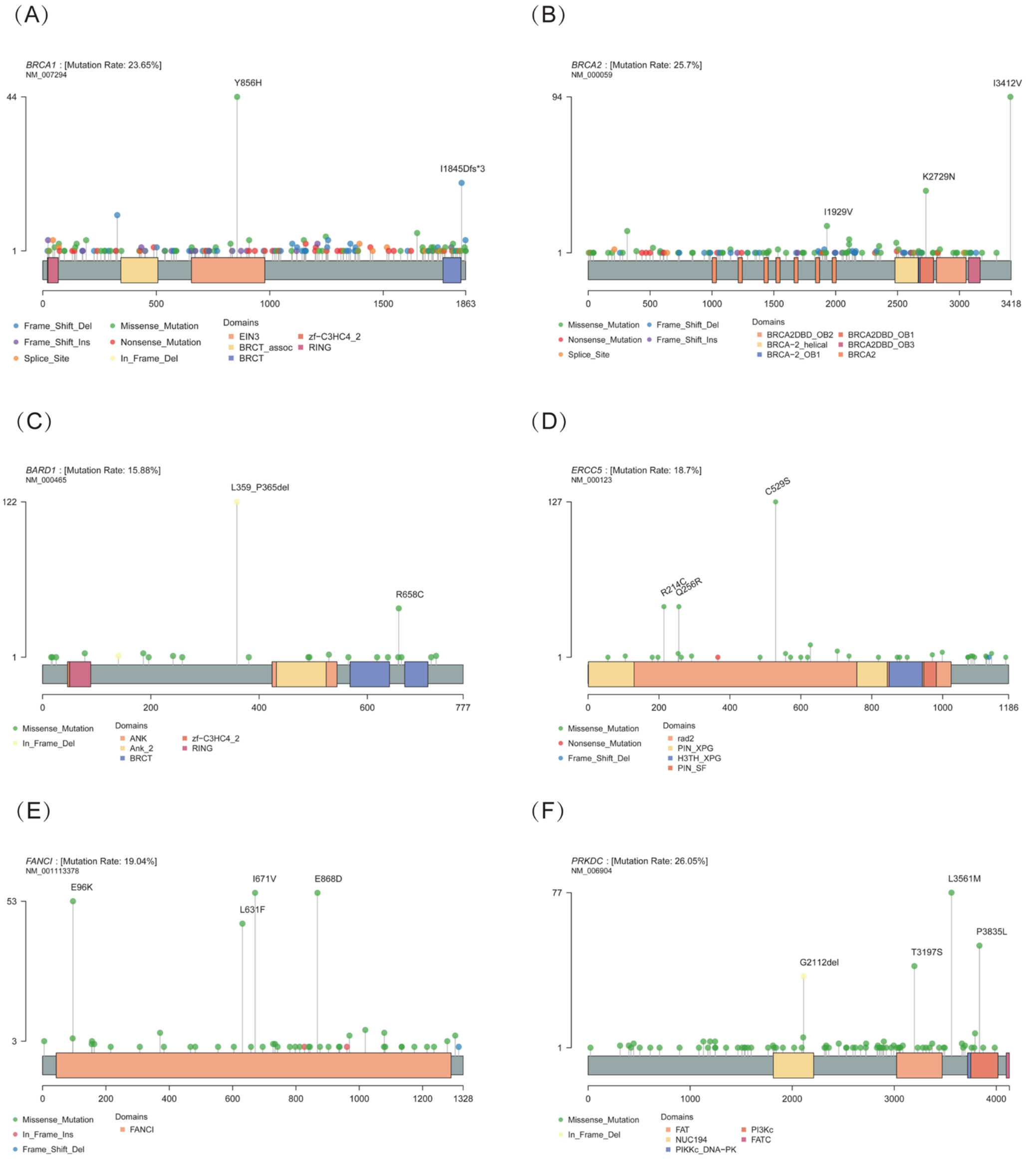

Supplement: Supplementary file 5 [file Image_10.pdf]
