# Supplementary material for: Mutation characteristics of cancer susceptibility genes in Chinese ovarian cancer patients
Source: Front Oncol. 2024 May 16;14:1395818. doi: 10.3389/fonc.2024.1395818 (PMC11137316; doi:10.3389/fonc.2024.1395818)
Supplement: Supplementary Material 1 — Mutation detection methods. [file Presentation_1.pdf]

Supplementary Material 1 Mutation detection methods

Next-generation sequencing

Blood samples were collected at hospital and sent to BGI Shenzhen Clinical Diagnostic Laboratory, where DNA extraction, targeted DNA sequencing, variant calling, and interpretation were performed[1]. Briefly, genomic DNA (gDNA) was extracted from participants' peripheral blood using the Qiagen Blood Midi Kit (Qiagen, Hilden, Germany) according to the manufacturer's standard protocol. DNA concentration and quality were assessed by Qubit (Life Technologies, Carlsbad, USA) and agarose gel electrophoresis. The gDNA (250ng) was randomly fragmented by the Covaris LE220 sonicator (Covaris, Woburn, USA) to generate gDNA fragments with a peak of 250 bp and then subjected to three enzymatic steps: end-repair, A-tailing, and sequencer (MGI, Shenzhen, China) adapter ligation. DNA libraries were purified with Agencourt Ampure XP beads (Beckman-Coulter, Indiana, USA), and PCR was carried out to form a pre-PCR library or pre-hybridization library, during which a unique 8 bp barcode was added to label each sample. Five to ten pre-PCR libraries were pooled equally and hybridized to a custom hereditary cancer panel (BGI, Shenzhen, China), which contained 171 genes. After purification, the enriched DNA was specifically captured and amplified by PCR to obtain a post-PCR library. The post-PCR library was subjected to single-strand separation, circularization and rolling circle replication to generate DNA nano balls (DNB) and sequencing was performed with 2 × 101 bp paired-end reads on a BGISEQ-500 or MGISEQ-2000 platform (MGI, Shenzhen, China) following the manufacturer's protocols. SNVs and INDELs in all coding exons and intron-exon boundaries (±20 base pairs) of detected genes were identified from NGS data.

Sequencing data analysis

Raw fastq data generated by the sequencer was first filtered by SOAPnuke 1.5.0 to exclude low quality reads[2]. The clean reads were then aligned to the reference human genome (UCSC hg19) using the Burrows-Wheeler Aligner (BWA 0.7.12) MEM algorithm[3]. PCR deduplication was performed using Picard 1.87. The average depth was over 100X and the coverage at 30X exceeded 95% for each sample. Single-nucleotide variants (SNVs), small insertions and deletions (INDELs) were detected by Genome Analysis Toolkit (GATK 4.0.8.1) HaplotypeCaller[4]. SNVs and INDELs in all coding exons and intron-exon boundaries (±20 base pairs) of detected genes were identified from NGS data. All of above variants were further filtered by quality depth, strand bias, mapping quality and read position. Finally, each variant was annotated by Bcfanno for gene location and predicted function in Human Genome Variation Society (HGVS) nomenclature and was ready for interpretation.

Reference

1. Yin RT, Chen JH, Li QL, Yan XY, Wang J, Li KM, Song L, Zhong L, Ma Y, Zhang MP, Zeng J, Wang DQ, et al. Characteristics of germline DNA damage response gene mutations in ovarian cancer in Southwest China. *Journal of Clinical Oncology* 2022;**40**: E17606-E.
2. Chen Y, Chen Y, Shi C, Huang Z, Zhang Y, Li S, Li Y, Ye J, Yu C, Li Z, Zhang X, Wang J, et al. SOAPnuke: a MapReduce acceleration-supported software for integrated quality control and preprocessing of high-throughput sequencing data. *Gigascience* 2018;**7**: 1-6.
3. Li H, Durbin R. Fast and accurate short read alignment with Burrows-Wheeler transform. *Bioinformatics* 2009;**25**: 1754-60.
4. Van der Auwera GA, Carneiro MO, Hartl C, Poplin R, Del Angel G, Levy-Moonshine A, Jordan T, Shakir K, Roazen D, Thibault J, Banks E, Garimella KV, et al. From FastQ data to high confidence variant calls: the Genome Analysis Toolkit best practices pipeline. *Curr Protoc Bioinformatics* 2013;**43**: 11 0 1- 0 33.
