# Supplementary material for: Mutation characteristics of cancer susceptibility genes in Chinese ovarian cancer patients
Source: Front Oncol. 2024 May 16;14:1395818. doi: 10.3389/fonc.2024.1395818 (PMC11137316; doi:10.3389/fonc.2024.1395818)

Figure S5. Comparison of BRCA1 and BRCA2 high-frequency mutations in the enrollment cohort and the detection frequency of GnomAD database

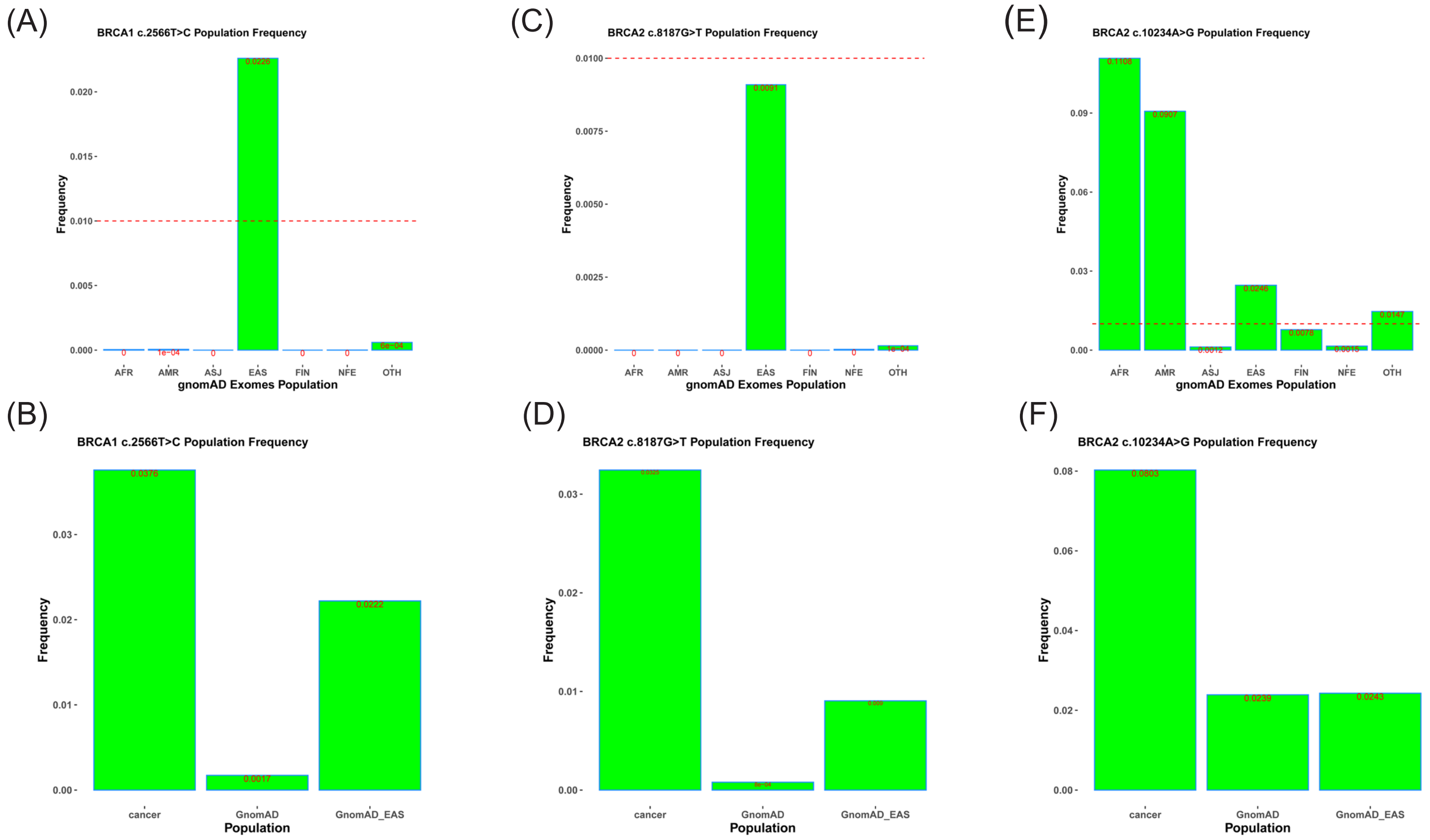

Supplement: Supplementary Figure 8 — Differences in mutation genes among the different age. (A) The distribution of number of sample mutations at different ages. (B) Differences in mutation genes between the younger group and the older group. [file Image_5.pdf]
